# Fructose and lactose intolerance and malabsorption testing: the relationship with symptoms in functional gastrointestinal disorders

**DOI:** 10.1111/apt.12306

**Published:** 2013-04-09

**Authors:** C H Wilder-Smith, A Materna, C Wermelinger, J Schuler

**Affiliations:** Gastroenterology Group Practice, Brain-Gut Research GroupBern, Switzerland

## Abstract

**Background:**

The association of fructose and lactose intolerance and malabsorption with the symptoms of different functional gastrointestinal disorders (FGID) remains unclear.

**Aim:**

To investigate the prevalence of fructose and lactose intolerance (symptom induction) and malabsorption and their association with clinical gastrointestinal (GI) as well as non-GI symptoms in FGID and the outcome of dietary intervention.

**Methods:**

Fructose and lactose intolerance (defined by positive symptom index) and malabsorption (defined by increased hydrogen/methane) were determined in 1372 FGID patients in a single centre using breath testing. Results were correlated with clinical symptoms in different FGID Rome III subgroups. The effectiveness of a targeted saccharide-reduced diet was assessed after 6–8 weeks.

**Results:**

Intolerance prevalence across all FGIDs was 60% to fructose, 51% to lactose and 33% to both. Malabsorption occurred in 45%, 32% and 16% respectively. There were no differences in intolerance or malabsorption prevalence between FGID subgroups. FGID symptoms correlated with symptoms evoked during testing (*r* = 0.35–0.61. *P* < 0.0001), but not with malabsorption. Non-GI symptoms occurred more commonly in patients with intolerances. Methane breath levels were not associated with constipation using several cut-off thresholds. Adequate symptom relief was achieved in >80% of intolerant patients, irrespective of malabsorption.

**Conclusions:**

Fructose and lactose intolerances are common in FGID and associated with increased non-GI symptoms, but not with specific FGID subtypes. Symptoms experienced during breath testing, but not malabsorption, correlate with FGID symptoms. Effective symptom relief with dietary adaptation is not associated with malabsorption. Mechanisms relating to the generation of GI and non-GI symptoms due to lactose and fructose in FGID need to be explored further.

## Introduction

Adverse reactions to food are common in the population and are claimed by up to 67% of individuals with Functional Gastrointestinal Disorders (FGID).^1^ They form part of the Rome III definition of Functional Dyspepsia (FD), and are frequent in Irritable Bowel Syndrome (IBS).^2^ Food hypersensitivity is often difficult to confirm, but avoidance of specific foods often diminishes symptoms. Possible underlying mechanisms include nutrient maldigestion or malabsorption, chemical or mechanical hypersensitivity, changes in gastrointestinal motility, the enteric microbiome, and immune and psychological responses. Reliable tests are lacking for several of these mechanisms and results may be ambiguous, for example, for tests of malabsorption and allergy. However, there has been recent progress, both in more refined test methodology and in interventional studies.^3^, ^4^ Although carbohydrate intolerances, defined as symptoms associated with their ingestion, are probably not the cause of most FGID, the reduced consumption of fermentable saccharides in patients with malabsorption results in symptom relief superior to most pharmaceutical treatments.^5^, ^6^ However, the significance of carbohydrate-related symptoms and of malabsorption in FGID remains unclear, as do the optimal diagnostic techniques.^7^ Furthermore, the common association of GI intolerances with non-GI reactions in FGID remains unexplained.

In this study, the following issues were examined in a large single-centre cohort of successive FGID patients: (i) How common are lactose and fructose intolerance and malabsorption during breath testing in FGID and its subgroups? (ii) What is the relationship between clinical symptoms and breath test results? (iii) Are non-GI symptoms associated with intolerances? and (iv) What is the symptomatic outcome of a standardised dietary intervention and is this related to malabsorption? Our hypotheses were that fructose and lactose intolerance would be equally common across all FGID, that symptoms generated during breath testing would correlate with the patients' clinical symptoms and that intolerance would be more relevant than only malabsorption in the diagnosis and dietary treatment outcome of fructose and lactose intolerance. We expected a high incidence of non-GI symptoms in patients with intolerances.

## Materials and methods

All successive patients referred to our gastroenterology practice by general practitioners between January 2008 and May 2011 for evaluation of FGID were eligible for inclusion in this prospective study, except those with evidence of organic disease, which was assessed by routine haematology and biochemistry blood testing and also stool testing for calprotectin and pancreas elastase determined in two stool samples in all patients. Parasite and bacterial stool cultures were performed if clinically indicated. Upper and lower endoscopies with biopsies were required in patients older than 40 years or in patients with diarrhoea or faecal blood.^8^ Coeliac disease was excluded by antitranglutaminase antibodies or duodenal biopsies. One consultant gastroenterologist (CWS) performed all the medical and dietary history taking and physical examinations. The dietary history included two sections: an open question requesting a listing of avoided and poorly tolerated foods and then a specific list of the main fructose, fructooligosaccharide, galactosaccharide, lactose and sorbitol-containing foods as well as the 10 commonest food allergies in Europe. In addition, skin rashes, urticaria, rhinitis, headache, imperative defaecatory urge, changes in stool consistency related to mealtimes were documented. All patients completed a standardised questionnaire, which included the specific questions for classification of GI symptoms into FGID groups according to the Rome III criteria and additional questions regarding allergies, childhood and family history, central nervous, musculoskeletal and cardiac system symptoms, and the use of polyol-containing sweets and chewing gum. Patients were classified into FGID subgroups according to the Rome III criteria.^9^ The most prominent FGID was chosen for classifying each patient. The study was performed in accordance with the Helsinki Declaration of 1975 as revised in 1983.

### Breath test protocol

Fructose and lactose breath tests were performed in all FGID patients by one laboratory technician (AM). No antibiotics, colonoscopy or laxatives were permitted within 14 days and a specific low-saccharide diet was adhered to 1 day before the tests. Patients arrived for testing in the morning after fasting overnight and without having smoked, chewed gum or performed vigorous exercise for at least 4 h. Chlorhexidine mouthwash was used and teeth were brushed before testing. The breath tests were performed in randomised, patient-blinded sequence on two separate occasions at least 4 days apart. Breath samples were collected in sealed glass tubes (Quintron Instruments, Milwaukee, WI, USA) before and hourly for 5 h after ingestion of lactose 50 g or fructose 35 g dissolved in 300 mL water. Hydrogen, methane and CO_2_ concentrations were measured within 72 h using the Quintron BreathTracker SC® (Quintron Instruments). Hourly testing was chosen based on our laboratory pilot data, where identical qualitative results were obtained as with sampling every 15 min and concentrations were stable in the tubes for 3 weeks. Malabsorption was defined as an increase of >20 ppm in hydrogen or >10 ppm in methane levels over baseline twice in succession. The numbers of patients with early rises (in the first 60 min) above the 20 ppm threshold in breath hydrogen concentration following fructose and lactose were calculated for comparison with other published data^7^, ^10^–^16^ Intolerance was defined as an increase of >2 over baseline using a symptom score index, which was the sum of the intensities (0 = none, 1 = mild, 2 = intense) of abdominal distension or bloating, flatulence, fullness, nausea, diarrhoea, abdominal cramps, borborygmi and gastro-oesophageal reflux symptoms, which were scored hourly concurrently with the collection of the breath samples. Additional non-GI symptoms rated, but not part of the symptom index, were tiredness, diminished concentration, headache, myalgia, arthralgia, palpitations, oral aphthoid ulcers and skin rash. The choice of symptoms scored was based on the literature and the most frequent atopic and co-morbid functional disorders.^6^, ^12^, ^17^

Additional methane threshold definitions for malabsorption of >3 ppm at baseline and an increase of >20 ppm over baseline at any time during the test were applied for correlations with stool patterns for comparison with previous publications.^18^ Diarrhoea was defined as loose or watery stools during >25% of bowel motions or >3 motions daily in the last 3 months. Constipation was characterised by hard, lumpy stools and increased straining during >25% of bowel motions or <3 stools per week in the last 3 months.

### Dietary protocol

The 312 patients with positive intolerance tests after May 2010 were referred to an experienced dietician (CW) for a standardised 4-week dietary adaptation, consisting of a diet low in saccharides and polyols for 1 week and subsequent weekly introduction of defined classes and amounts of fructose-, fructan-, inulin- and lactose-containing food to determine individual tolerability thresholds. Patients were maintained on the level of saccharides and polyols below their threshold of symptoms. In general, four individual sessions were scheduled with patients and questionnaires regarding abdominal symptoms, bowel and dietary habits were completed before and after the dietary modification. Symptom scoring was performed using 10-point Likert scales. Dietary compliance was checked either by direct or telephonic interview by the dietician or the gastroenterologist 6–8 weeks after initiation of the dietary changes. Compliance was considered adequate if patients confirmed that they adhered to the dietary guidelines during at least 50% of the meals consumed.^19^

### Statistics

Group differences in clinical GI and non-GI symptoms and variables were compared by Kruskall–Wallis or anova tests, as appropriate. Categorical data were compared by Chi-squared test or, if between multiple groups, by logistical regression. Correlations were analysed using the Spearman–Rank test. A significance threshold of *P* < 0.05 was adopted. Analysis was performed using Statistica 7.1 (StatSoft, Tulsa, OK, USA).

## Results

Patient characteristics are shown in Table 1. Seventy-six per cent of the 1372 patients were of Northern European and 19% of Mediterranean European Caucasian descent. Of these patients, the following subgroups were defined according to the Rome III criteria: Irritable Bowel Syndrome (*n* = 212), comprised of IBS with constipation (IBS-c, *n* = 37), IBS with diarrhoea (IBS-d, *n* = 67), IBS with alternating constipation and diarrhoea (IBS-m, *n* = 94) and IBS unclassified (IBS-u, *n* = 14), Functional Dyspepsia(*n* = 606), comprised of FD with postprandial distress (FD-ppd, *n* = 368) and FD with epigastric pain syndrome (FD-eps, *n* = 238), and Functional Bloating (*n* = 109).

**Table 1:** Patient characteristics. There are no significant differences in the main characteristics or demographics between the groups of functional GI disorders

	All functional GI disorders	Irritable bowel syndrome†	Functional dyspepsia†	Functional bloating†
Number of patients	1372	212	606	109
Age (years)*	42 ± 16	38 ± 16	40 ± 13	48 ± 15
Gender (% female)	73	77	73	62
Symptom duration (years)*	5.9 ± 5	7.2 ± 6	5.2 ± 5	5.6 ± 6
BMI (kg/m^2^)*	23.5 ± 5	24.1 ± 4	23.4 ± 5	22.1 ± 6

* Mean ± s.d.

† Subgroups of functional GI disorders as defined by the Rome III criteria.^9^

### Prevalence of lactose and fructose intolerance during testing and association with clinical symptoms

**Figure 1:**
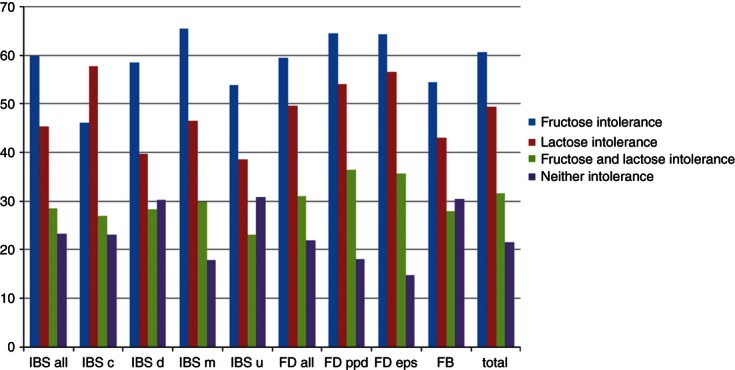
Prevalence of fructose and lactose intolerances in Rome III-defined Irritable Bowel Syndrome (IBS) (*n* = 212), Functional Dyspepsia (FD) (*n* = 606), Functional Bloating (FB) (*n* = 109) and their subgroups, IBS-c: IBS with constipation (*n* = 37), IBS-d: IBS with diarrhoea (*n* = 67), IBS-m: IBS with alternating constipation and diarrhoea (*n* = 94), IBS-u: unclassified IBS (*n* = 14), FD-ppd: Functional Dyspepsia with postprandial distress (*n* = 368), FD-eps: Functional Dyspepsia with epigastric pain syndrome (*n* = 238) and FB: Functional Bloating (*n* = 109).

The prevalence of fructose, lactose and both intolerances in all FGID patients was 60.4%, 50.5% and 33.1% respectively. In Northern European and Mediterranean Caucasians, 61.0% and 59.4% had fructose intolerance (not significant-N.S.), 47.8% and 53.6% had lactose intolerance (*P* < 0.01), and 33.2% and 29.5% had both intolerances (N.S.). Of males and females, 57.1% and 61.7% had fructose intolerance (N.S.), 43.6% and 53.3% had lactose intolerance (*P* < 0.01), and 27.6% and 35.2% had both intolerances (*P* < 0.05). Fructose was more common than lactose intolerance in all FGID subgroups (*P* < 0.001)(Figure 1), with no differences between FGID groups, except for more lactose and less fructose intolerance in constipated IBS (IBS-C) than in other subgroups (*P* < 0.05). 14.1% of patients with lactose intolerance and 6.9% of patients with fructose intolerance correctly suspected the target of their intolerance before breath testing.

The prevalence of non-GI and specific GI symptoms and related aspects of the medical history were compared across intolerance groups (Table S1). Overall, patients with any intolerance had more symptoms than those without (*P* < 0.001). Patients with both intolerances had significantly more problems with concentration, joint and muscle pain, nausea, gastro-oesophageal reflux and allergic reactions than those with no intolerances and generally also than patients with only one intolerance (Table S1). When comparing patients with either fructose or lactose intolerance with patients with no intolerances, very similar, albeit less prominent, differences emerged (Table S1).

**Figure 2:**
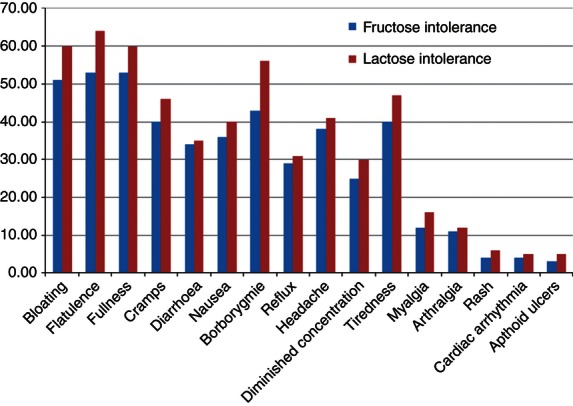
Percentages of patients with Functional Gastrointestinal Disorders (FGID) with symptoms provoked during fructose or lactose breath testing (*n* = 1372).

The incidence of symptoms *during fructose and lactose breath tests* is shown in Figure 2. Flatulence, bloating and abdominal fullness were predominant in >50% of patients. Fifty-eight per cent of patients complained of at least one central nervous system effect, with fatigue topping the list. Symptom incidences were similar between both intolerance tests. The time-to-maximum symptom score was 115.3 ± 118 min with fructose and 131.1 ± 118 min with lactose (*P* < 0.001). Using a test cut-off time of 3 h instead of 5 h, 16% of fructose and 23% of lactose intolerance results would have changed from positive to negative.

**Figure 3:**
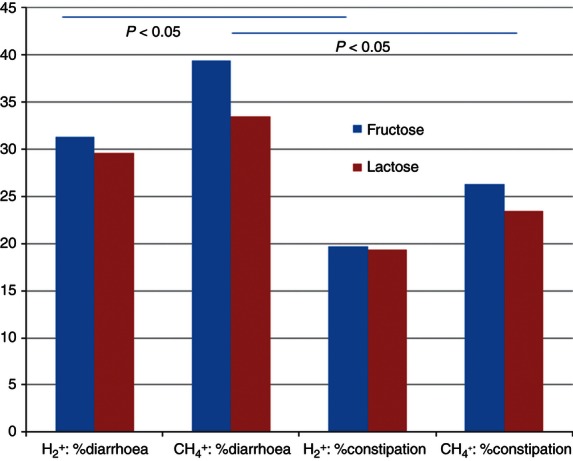
Percentages of patients with Functional Gastrointestinal Disorders (FGID) with fructose (*n* = 613) or lactose (*n* = 432) malabsorption, i.e. an increase of H_2_ > 20 ppm or CH_4_>10 ppm over baseline, with diarrhoea or constipation. *P < 0.05 constipation vs. diarrhoea for both fructose and lactose.

The incidence of several of the most commonly evoked symptoms during breath testing, specifically bloating, diarrhoea, abdominal pain, gastro-oesophageal reflux, tiredness, muscle pain and diminished concentration, correlated with patients' FGID symptoms (*r* = 0.34–0.51, all *P* < 0.005).

### Prevalence of lactose and fructose malabsorption and the relationship with intolerance during testing and clinical symptoms

Table 2 shows the prevalence and overlap of malabsorption and intolerance in FGID and its subgroups. Malabsorption alone, i.e. with a negative intolerance test, occurred in 4.3% of fructose, 3.4% of lactose and 3% of both tests. Intolerance alone, i.e. with a negative malabsorption test, was seen in 20.0% of fructose, 22.4% of lactose and 20.2% of both tests, with no significant differences between the FGID subgroups.

**Table 2:** Percentages of patients with Irritable Bowel Syndrome, Functional Dyspepsia, Functional Bloating, classified according to Rome III criteria^9^ and of all Functional GI Disorder (FGID) patients with intolerance (increase in symptom scoring >2), malabsorption (increases >20 ppm of H_2_ and/or >10 ppm of CH_4_ above baseline) or both intolerance and malabsorption during fructose and lactose testing. There are no differences in the prevalence of intolerance or malabsorption between the groups of functional disorders. Intolerance is significantly more common than malabsorption in all groups

	Intolerance (%)	Malabsorption (%)	Intolerance and malabsorption (%)
Fructose			
Irritable Bowel Syndrome *n* = 212	59.9*	42.4	38.4
Functional dyspepsia *n* = 606	59.5*	41.6	37.2
Functional bloating *n* = 109	54.4*	41.8	39.2
All FGID *n* = 1372	60.4*	44.7	40.4
Lactose			
Irritable Bowel Syndrome *n* = 212	45.3*	29.1	25.0
Functional dyspepsia *n* = 606	49.7*	30.8	27.8
Functional bloating *n* = 109	43.0†	32.9	26.6
All FGID *n* = 1372	50.5*	31.5	28.1
Fructose and Lactose			
Irritable Bowel Syndrome *n* = 212	28.5*	14.0	11.0
Functional dyspepsia *n* = 606	31.0*	15.4	12.6
Functional bloating *n* = 109	27.8*	16.5	12.7
All FGID *n* = 1372	33.1*	15.9	12.9

* *P* < 0.01 or † *P* < 0.05 intolerance vs. malabsorption and vs. intolerance & malabsorption.

Malabsorption was most frequently defined by a combination of supra-threshold H_2_ and CH_4_ concentrations (Table 3). Isolated elevation of CH_4_ was seen in 4.1% of patients following fructose and in 4.9% after lactose ingestion using the standard threshold of a CH_4_ increase of >10 ppm above baseline. Table 3 shows malabsorption rates using alternative CH_4_ thresholds.

**Table 3:** The frequency (%) of positive hydrogen (>20 ppm increase over baseline) or methane (>10 ppm increase over baseline) breath tests in patients with Functional GI Disorders with malabsorption after ingestion of fructose 35 g or lactose 50 g. Additional results for methane-only producers are shown using the thresholds of methane >20 ppm over baseline at any time during the test, as well as a baseline concentration >3 ppm. The coincidence of increased hydrogen and methane is the most common constellation for malabsorption. The definition of a methane producer is markedly influenced by the threshold chosen

Breath test results defining malabsorption	Fructose (%)	Lactose (%)
Thresholds of H_2_ > 20 ppm and CH_4_ > 10 increase	*n* = 613	*n* = 432
H_2_ + CH_4_−	29.0	21.6
H_2_ + CH_4_ +	66.8	74.4
H_2_−CH_4_ +	4.1	4.9
Alternative thresholds of CH_4_		
H_2_−CH_4_ + (>20 ppm increase)	1.2	1.1
H_2_−CH_4_ + (baseline >3 ppm)	18.0	21.3

H_2_ and CH_4_ peak concentrations correlated with the severity of bloating (*r* = 0.41, *r* = 0.43 respectively), abdominal pain (*r* = 0.35, *r* = 0.33 respectively) and diarrhoea (*r* = 0.5, *r* = 0.45 respectively)(all *P* < 0.001) during fructose and lactose (*r* = 0.53, *r* = 0.52; *r* = 0.48, *r* = 0.48; and *r* = 0.61, *r* = 0.59, respectively, all *P* < 0.0001) testing. The time-to-peak H_2_ and CH_4_ concentrations were 107.7 ± 71 min and 107.3 ± 76 min with fructose, and 164.2 ± 105 min and 168.5 ± 106 min with lactose respectively (both *P* < 0.001 vs. fructose). Peak concentrations of H_2_ and CH_4_ were reached after 3 h in 10.1% and 37.7% of fructose tests and in 9.8% and 33.8% of lactose tests respectively. There were no significant correlations between H_2_ and CH_4_ peak concentrations and any FGID symptoms. Peak concentrations of H_2_ >20 ppm in the first 60 min, so-called early rises in breath hydrogen, were seen in 12.9% of patients following fructose and in 6.0% of patients following lactose ingestion.

### Breath gases and stool patterns

In patients with fructose or lactose malabsorption, as defined by any of the H_2_ or CH_4_ thresholds, diarrhoea was more common than constipation (all *P* < 0.05) (Figure 3). Conversely, patients with diarrhoea and constipation had a similar prevalence of malabsorption demonstrated by isolated elevation of H_2_ (14.5% and 11.3%) and CH_4_ (1.2% and 1.2%) concentrations following fructose and lactose ((7.7% and 6.5%) and (1.4% and 1.6%)) respectively. Furthermore, a majority (69%) of IBS-C patients did not have elevated CH_4_ levels after either sugar.

### Outcome of dietary advice

Complete outcomes were available in 237 of the 312 patients (76%) with intolerances who received standardised dietary counselling once it was initiated. Thirty-six patients declined dietary counselling and 39 dropped out of the dietary programme and were lost to follow-up. Clinical characteristics did not differ between those having discontinued or completed the programme. Adequate symptomatic relief after 6–8 weeks was achieved in 84% of all patients with fructose intolerance and in 86% when fructose malabsorption was present with intolerance. Respective results for lactose intolerance were 89% and 90%. Eight-five per cent of all patients reported adequate dietary compliance. Adequate relief rates were 85% and 96% in patients with diarrhoea and bloating, respectively, compared to 51% in those patients with constipation (*P* < 0.01). Symptomatic relief >3 on the 10-point scale was achieved in 90% of all fructose and 94% of all lactose intolerant, and in 93% and 96% of those with concurrent malabsorption, respectively. Average symptom relief was between 6 and 7 on the 10-point scale for all the above subgroups, except in constipated patients where the average was 3 (data not shown).

## Discussion

Both fructose and lactose intolerance as shown by breath testing were common in this large group of FGID patients, one third of whom had an overlap of both intolerances. The important question is whether intolerances are an underlying mechanism or an epiphenomenon in FGID. The prevalence of intolerances was similar across all major types of FGID, except IBS-C. As the different FGID phenotypes were not related to distinct distributions of the intolerances, a causal relationship between FGID and the intolerances would have to be explained by divergent host responses to the saccharide ingestion. Such potential host factors include the enteric microbiome, intestinal permeability, nervous system and immune responses, all of which are interrelated and differ between FGID and controls.^20^–^24^ Hypersensitivity to distension and to ingested nutrients has been shown in different FGID, but we are not aware of comparisons between subgroups, except in IBS-C, where fermentation processes may be influenced by a prolonged transit time.^16^, ^25^–^31^

The induction of symptoms (intolerance) following sugar ingestion appears to be more relevant than malabsorption *per se* in FGID, as demonstrated by the following observations. Patients' main clinical GI and non-GI symptoms were significantly reproduced during breath testing, but there was no association with the markers of malabsorption. Malabsorption is similarly common after fructose or lactose loading in IBS and controls, but symptom induction is much higher in IBS.^7^, ^12^, ^13^, ^32^, ^33^ Furthermore, in patients with intolerance, effective symptom relief with dietary adaptation was independent of the presence of malabsorption. The relationship between malabsorption and intolerance therefore appears to be indirect. Intolerance without evidence of malabsorption occurred in approximately 20% of our patients and in 18–40% in earlier studies, whereas malabsorption rarely existed without intolerance.^6^, ^34^ Potential explanations for this discrepancy are a microbiome not producing measurable levels of hydrogen or methane in some individuals or an alternative mechanism for symptom generation, such as increased chemosensitivity to fermentation products.^35^–^37^ Increased production of sulphide from carbohydrate fermentation has been shown in IBS.^38^ It should be noted that the relationship between symptom induction, and therefore intolerance rates, and malabsorption is dependent on the choice of symptom scoring, with a positive association between the number and intensity of induced symptoms and the percentage of positive hydrogen breath tests following lactose having been shown.^38^ In this study, a sensitive symptom index reflecting both intensity and symptom number was used very similar to the 9-item validated score used by Choi *et al*., with intolerance rates not greater than in other large comparative studies in Caucasians for lactose – we are not aware of any similar large studies for fructose intolerance.^6^, ^39^ Only a small minority of these referred patients were able to correctly identify their food intolerance before the provocation testing, but this undoubtedly also partly reflects a referral bias to a GI practice.

Dietary modification based on fructose and lactose intolerance testing was clearly beneficial. Over 80% of all FGID patients attained adequate global symptom relief and average relief was 7 on the 10-point symptom scale. Adequate symptom relief was lower at about 50% in patients with constipation. These data are consistent with an earlier study where 85% of 48 IBS patients compliant with a similar diet reported an improvement of >5 on the 10-point scale for pooled symptoms after 14 months and also a reduced response rate in constipated patients.^40^ The impressive responses in this and smaller open trials in FGID are confirmed by a double-blinded, placebo-controlled fructose and fructan challenge study.^5^, ^6^, ^40^–^42^ Psychological and disease fluctuation components in our response rates could only be assessed in a blinded and placebo-controlled dietary study, which was not feasible in a study of this scale due to the inherent complexity.

Non-GI co-morbidity was common in FGID, especially in the central nervous, musculoskeletal and atopic categories. An elevated prevalence of non-GI functional syndromes, including fibromyalgia, migraine and chronic fatigue, is reported in FGID.^43^, ^44^ Over 50% of our patients reported CNS symptoms, with fatigue being most frequent. Tiredness has previously been associated with a high FODMAP diet in IBS patients.^45^ Non-GI symptoms in FGID patients progressively increased with the number of experienced intolerances. The mechanisms underlying the association between GI and non-GI symptoms were not investigated, but they may be central (e.g. somatisation, central sensory processing), peripheral (e.g. afferent sensory sensitisation) or both (e.g. neuro-immune activation, disseminated abnormality in transporter proteins). The spectrum of non-GI symptoms was similar in fructose and lactose intolerances, except for more joint pain, prandial rhinitis and reactions to cosmetics with fructose intolerance. Earlier, small studies demonstrated increased depression, lethargy, decreased plasma tryptophan and various trace elements in fructose intolerance and a range of non-GI symptoms in lactose intolerance, including cardiac arrhythmias, musculoskeletal, atopic and nervous system symptoms.^46^–^50^ Intriguing potential links include toxic metabolites produced by anaerobic digestion, accumulation of advanced glycation end-products, abnormalities in fructose transporter proteins, increased intestinal permeability, actions of the phlorizin hydrolase moiety of lactase and enzyme homologies with inflammatory mediators, none of which have been confirmed. The short-chain fatty acids derived from colonic fermentation of fructose and lactose are similar.^51^ Further evaluation with specific tools is clearly warranted.

The epidemiology of fructose intolerance is poorly characterised. In our FGID patients, fructose was more common than lactose intolerance and more frequent in Northern than Southern Europeans, in contrast to lactose intolerance.^16^ Intolerances were more prevalent in females, significantly so for lactose, but confirmation is required in a population-based study. Interestingly, the overall prevalence of intolerances was higher in all FGID patients than in the more narrowly defined Rome III FGID subgroups. Consequently, studies employing Rome III criteria are likely to underestimate the overall relevance of intolerances.

The optimal breath test methodology remains unclear despite attempts at standardisation.^10^, ^11^ In this study, average maximum symptom scores were attained 15 min earlier following fructose than lactose. Using the popular 3-h test duration, 16% of fructose and 23% of lactose intolerance results would have changed from positive to negative. H_2_ and CH_4_ concentrations peaked over 50 min earlier following fructose than lactose, and after 3 h in approximately 10% and 35% of tests respectively. The earlier exhaled gas and symptom peaks with fructose confirm an earlier report and may be due to differences in the location of absorption/digestion or of bacterial metabolism.^35^ The definitions of the gas concentration thresholds for a positive breath test are the subject of debate, demonstrably affecting test interpretation.^7^, ^13^ We used the most widely reported dose of lactose (50 g) and a fructose dose (35 g) in the range of daily consumption in Europe, inducing GI symptoms in <10% of controls, and allowing comparison with seminal FODMAP studies.^3^, ^5^, ^7^, ^14^, ^32^, ^35^, ^52^ Both of these sugar doses are higher than the average amounts of free saccharide ingested during a single meal in normal life, but are used as provocation tests to identify patients likely to benefit from dietary manipulation. On the basis of the above, we recommend a 5-h test duration and, in case of discrepancy between symptom and malabsorption data, to base the final test interpretation on the intolerance results. This recommendation will result in effective treatment of 20% more patients compared with current practice in centres where clinical decisions are based exclusively on malabsorption. Furthermore, the correlation between clinical symptoms and those experienced during breath testing is an important confirmation for the patient. A breath collection duration longer than 3 h has been recommended in previous studies for improved diagnostics of lactose intolerance.^17^, ^39^

This study provides no corroboration for an association between exhaled methane and constipation using any of the common CH_4_ threshold definitions.^18^ Diarrhoea was more common than constipation in patients with elevated breath CH_4_. Conversely, increased CH_4_ was equally prevalent in constipation and diarrhoea. Furthermore, in IBS-C, breath tests were significantly more frequently CH_4_ negative than positive. This discrepancy with previous publications may be explained by variations in the definition of constipation (stool characteristics, transit, manometric changes, global evaluation), test methodology (CH_4_ threshold defined by very low baseline or peak concentrations, test duration, type of sugar) or patient selection (IBS, functional constipation, proportion with diarrhoea). Further study is required given this wide heterogeneity and the positive associations described mainly in smaller subgroups.

The relationship and overlap between small intestinal bacterial overgrowth (SIBO) and sugar malabsorption is extensive and it is acknowledged that currently a reliable distinction is not possible.^53^ Indeed, both conditions are intricately interrelated and the fermentation of sugars reaching bacteria in the colon (malabsorption) or in the small intestine (SIBO) can register similarly in breath tests, depending on the underlying rapidity of intestinal transit and the composition of the microbiome, among other factors. Different constructs for a distinction have been presented, such as early hydrogen peaks, gas thresholds and different substrates, but a true distinction remains elusive and we have chosen not to attempt one in our study. However, for a comparison with other papers, we have included the rather low percentage of patients with early rises in breath hydrogen excretion.

Study limitations are the absence of a blinded, placebo-controlled design with healthy controls. The choice of an inert placebo in this group of sensitive patients is challenging. Healthy controls were not included in this study aiming at comparisons within FGID. Comparisons with controls have been reported previously. An overlap between FGID subgroups is inevitable in all related studies. We chose the most prominent FGID for classifying each patient. Advantages of our single-centre study are the large number of successive patients with different FGID tested for both intolerance and malabsorption and treated in standardised fashion. Meta-analyses in this field are problematic due to the inherent variability in patient selection and in testing and treatment procedures.

In conclusion, fructose and lactose intolerance are common and frequently overlap in FGID, with no differences in prevalence between subgroups. Non-GI symptoms are more common in FGID patients with intolerances. Effective symptom relief is achieved with standardised dietary adaptation. Clinical FGID symptoms correlate with the symptoms induced during testing and not with malabsorption, which consequently does not appear to be the main driver of symptoms. Mechanisms relating to carbohydrate intolerance and accompanying non-GI symptoms are of special interest for future research.

## Authorship

*Guarantor of the article*: Clive H. Wilder-Smith.

*Author contributions*: Clive Wilder-Smith designed the research. Andrea Materna and Corinne Wermelinger conducted the research. Jonathan Schuler and Clive Wilder-Smith analysed the data or performed the statistical analysis. Clive Wilder-Smith wrote the paper and had primary responsibility for the final content. All authors read and approved the final version of the manuscript.

## SUPPORTING INFORMATION

**Table S1**. Prevalence (%) of clinical non-gastrointestinal and select gastrointestinal symptoms, and relevant aspects of the medical history according to fructose and lactose intolerance status. Intolerance is defined by an increase of >2 in symptom scoring following administration of the saccharide. Overall, the frequency of clinical symptoms and of a history of atopy was in the following order of magnitude in patients: both fructose and lactose intolerances > only one intolerance > no intolerances.
