# Supplementary material for: Fructose and lactose intolerance and malabsorption testing: the relationship with symptoms in functional gastrointestinal disorders
Source: Aliment Pharmacol Ther. 2013 Apr 9;37(11):1074–83. doi: 10.1111/apt.12306 (PMC3672687; doi:10.1111/apt.12306)
Supplement: Supplementary file 1 [file apt0037-1074-SD1.docx]

**Table S1.** Prevalence (%) of clinical non-gastrointestinal and select gastrointestinal symptoms, and relevant aspects of the medical history according to fructose and lactose intolerance status. Intolerance is defined by an increase of >2 in symptom scoring following administration of the saccharide. Overall, the frequency of clinical symptoms and of a history of atopy was in the following order of magnitude in patients: both fructose and lactose intolerances > only one intolerance > no intolerances.

|  | Percentages of patients in each group | | | | *p-values* | | | | | |
| --- | --- | --- | --- | --- | --- | --- | --- | --- | --- | --- |
| Intolerances: | Fructose^1^  (F)  N=829 | Lactose^1^ (L)  N=693 | Fructose & lactose^1^ (FL)  N=454 | None  (N)  N=303 | *F v L* | *F v FL* | *F v N* | *L v FL* | *L v N* | *FL v N* |
| Bloating | 49 | 48 | 50 | 52 | *NS* | *NS* | *NS* | *NS* | *NS* | *NS* |
| Problems with concentration | 41 | 41 | 47 | 34 | *NS* | *0.04* | *0.03* | *0.04* | *0.03* | *0.0005* |
| Fatigue | 39 | 37 | 41 | 37 | *NS* | *NS* | *NS* | *NS* | *NS* | *NS* |
| Abdominal pain | 36 | 36 | 40 | 34 | *NS* | *NS* | *NS* | *NS* | *NS* | *NS* |
| Arthralgia | 34 | 27 | 33 | 25 | *0.003* | *ns* | *0.004* | *0.03* | *ns* | *0.02* |
| Runny nose with meals | 32 | 28 | 34 | 24 | *0.09* | *ns* | *0.01* | *0.03* | *ns* | *0.004* |
| Urge diarrhoea | 32 | 27 | 30 | 27 | *0.03* | *ns* | *ns* | *ns* | *ns* | *ns* |
| Myalgia | 29 | 30 | 35 | 19 | *ns* | *0.02* | *0.0009* | *0.08* | *0.0004* | *0.00001* |
| Pollinosis | 28 | 30 | 33 | 21 | *ns* | *0.06* | *0.02* | *ns* | *0.004* | *0.0004* |
| Nausea | 25 | 24 | 28 | 14 | *ns* | *ns* | *0.0001* | *ns* | *0.0003* | *0.00001* |
| Adverse reaction: medicines | 24 | 25 | 26 | 18 | *ns* | *ns* | *0.03* | *ns* | *0.04* | *0.009* |
| Gastroesophageal reflux symptoms | 23 | 24 | 26 | 18 | *ns* | *ns* | *0.06* | *ns* | *0.03* | *0.009* |
| Perceived cardiac arrhythmias | 21 | 19 | 23 | 17 | *ns* | *ns* | *ns* | *ns* | *ns* | *0.06* |
| Skin rash | 18 | 17 | 17 | 21 | *ns* | *ns* | *ns* | *ns* | *ns* | *ns* |
| Asthma | 17 | 14 | 18 | 10 | *ns* | *ns* | *0.003* | *ns* | *0.003* | *0.002* |
| Animal hair allergy | 17 | 17 | 18 | 10 | *ns* | *ns* | *ns* | *ns* | *ns* | *ns* |
| Depressive mood | 17 | 17 | 19 | 16 | *ns* | *ns* | *ns* | *ns* | *ns* | *ns* |
| Adverse reaction: cosmetics | 14 | 18 | 20 | 8 | *0.03* | *0.005* | *0.007* | *ns* | *0.00001* | *0.00001* |
| Generalized pruritus | 13 | 14 | 14 | 15 | *ns* | *ns* | *ns* | *ns* | *ns* | *ns* |
| Bee/wasp sting allergy | 13 | 13 | 13 | 15 | *ns* | *ns* | *ns* | *ns* | *ns* | *ns* |
| Plant contact allergy | 10 | 9 | 12 | 7 | *ns* | *ns* | *ns* | *ns* | *ns* | *0.03* |
| Adverse reaction to latex | 8 | 7 | 12 | 3 | *ns* | *0.02* | *0.003* | *0.005* | *0.01* | *0.00001* |
| Adverse reaction: household product | 7 | 9 | 11 | 4 | *ns* | *0.01* | *0.06* | *ns* | *0.006* | *0.0005* |
| Daily use of sugar-free chewing gum | 35 | 30 | 37 | 19 | *0.04* | *ns* | *0.00001* | *0.01* | *0.0004* | *0.00001* |
| Positive family history of allergy | 34 | 34 | 35 | 30 | *ns* | *ns* | *ns* | *ns* | *ns* | *ns* |
| Positive childhood history of allergy | 28 | 27 | 29 | 21 | *ns* | *ns* | *0.02* | *ns* | *0.05* | *0.01* |

^1^Comparison of all effects in intolerance group vs group without intolerance: p<0.001. NS=not significant

Significant and trend p-values between groups as determined by ANOVA are shown.
